# Relationship between driver gene mutations and clinical pathological characteristics in older lung adenocarcinoma

**DOI:** 10.3389/fonc.2023.1275575

**Published:** 2023-11-01

**Authors:** Xia Liu, Guopeng Jiang, Xuefei Sun, Guangfeng Su, Xuan Zhang, Dan Shen, Na Yan

**Affiliations:** ^1^Department of Thoracic Surgery, Affiliated Hospital of Shandong University of Traditional Chinese Medicine, Jinan, Shandong, China; ^2^Key Laboratory of Digital Technology in Medical Diagnostics of Zhejiang Province, Dian Diagnostics Group Co., Ltd., Hangzhou, Zhejiang, China

**Keywords:** lung adenocarcinoma, geriatric patients, gene mutation, clinicopathological features, next-generation sequencing

## Abstract

**Objectives:**

Lung adenocarcinoma (LUAD) is the most common newly diagnosed malignant tumor in older people. As older patients age, organ function decreases, leading to increased adverse reactions to treatment. The epidermal growth factor receptor (EGFR) and anaplastic lymphoma kinase tyrosine (ALK) tyrosine kinase inhibitors (TKIs) therapy are more effective and well-tolerated than chemotherapy, while the rate of genetic testing and subsequent targeted treatment among older patients remains relatively low, the clinical benefit limitation for those patients. This study aims to investigate the mutation characteristics of LUAD diver gene and its relationship with clinicopathological features in older LUAD.

**Materials and methods:**

A total of 275 patients were diagnosed as LUAD and were over sixty years old. We utilized next-generation sequencing technology to detect and analyze gene mutations in postoperative tissue specimens, including *EGFR, KRAS, ALK, ROS1, RET, MET, BRAF, HER2, PIK3CA* and *NRAS*.

**Results:**

A total of 90.18% (248/275) of older LUAD patients experienced genetic mutations. The *EGFR* (192, 69.82%) had the highest mutation rate among ten genes, followed by *KRAS* (21, 7.64%), *MET* (21, 7.64%), *ERBB2* (15, 5.45%), *RET* (9, 3.27%), *ALK* (8, 2.91%), *ROS1* (8, 2.91%), *PIK3CA* (6, 2.18%), *BRAF* (5, 1.82%) and *NRAS* (1, 0.36%). We also found thirty patients (15.63%) with *EGFR* mutations also having other gene mutations. The *L858R* mutation and exon19 deletion were the predominant *EGFR* mutations, accounting for 84.90% of *EGFR*-mutated patients. In addition, fifty-one kinds of *EGFR* mutations were detected, distributed in the protein tyrosine kinase catalytic domain (43, 84.31%), cysteine enriched domain (4, 7.84%), receptor binding domain (3, 5.88%), and EGFR transmembrane domain (1,1.96%). Ten cases of gene fusion mutation were detected. Two rare partner genes, *PKHD1* (P60:R34) and *STK39* (R33:S11), were detected by *ROS1* gene fusion. *RET* gene fusion revealed a rare companion gene *KCND2* (R11:K2). The *EGFR* mutations were more prevalent in female, non-smoking patients (p < 0.05), and the *KRAS* mutations were more common in male and smoking patients (p < 0.01). In addition, the *BRAF* mutations were more likely to occur in the right lung (p < 0.05).

**Conclusion:**

Older LUAD populations exhibit diverse genetic mutations, which may also exist simultaneously. Simultaneous detection of multiple genes by NGS can accelerate and enhance targeted treatment benefits for older LUAD patients, ultimately improving their quality of life.

## Introduction

Lung adenocarcinoma (LUAD) is the most common pathological subtype in NSCLC, accounting for approximately 55% (1). Half of the LUAD patients are over 60 years old at the time of diagnosis, while a further 30% are over 70 years old, which are defined as older population (2). The older LUAD patients usually do not tolerate surgery. These patients are prone to heart disease, diabetes, or other primary diseases (3, 4). Therefore, choosing treatment methods with low toxicity, low side effects, and good tolerance in older LUAD patients is a critical clinical concern. With the development of precise diagnosis and treatment, more and more evidence suggests that targeted therapy guided by gene mutations has greatly improved treatment choices and survival benefits for NSCLC patients, including older LUAD patients (5–7). However, LUAD driver gene mutation vary from region to region, patient to patient, lifestyle to lifestyle, and test methodologies (8–10). This study retrospectively analyzed ten LUAD-related driving genes in 275 older LUAD patients and explored their correlation with clinicopathological indicators such as gender, smoking status, tumor location, maximum diameter, lymph node metastasis and others.

## Materials and methods

### Patient selection

In this retrospective study, the clinical data of the patients were collected from the sample database of thoracic surgery (**Supplementary Table S1**). Inclusive criteria: 1) Imaging confirmed measurable lesions; 2) Patients with cytological or pathological diagnosis of LUAD in our hospital or other hospitals; 3) Preoperative radiotherapy and chemotherapy were not performed; 4) Medical records were comprehensive and fully documented; 5) Patients should be over 60 years of age. Patients were excluded based on the following criteria: 1) Cytological or pathological diagnosis was not clear; 2) Patients with malignant tumors on other organs; 3) Patients with severe liver and kidney dysfunction; 4) Without genetic test results or incomplete case data.

### Sample preparation and DNA extraction

Specimens for gene mutation detection were obtained from formalin-fixed paraffin-embedded (FFPE) tissues after cytoreductive surgery. Tumor purity was determined by hematoxylin and eosin staining. The proportion of tumor cells in the sample should be at least 40%. The nucleic acid was extracted using the QIAamp DNA FFPE Tissue Kit following instructions provided by Qiagen in Dusseldorf, Germany. DNA concentration was measured by Qubit 3.0 (Thermo Fisher Scientific, Waltham, USA). We evaluated the distribution of nucleic acid fragment sizes using Qsep100 (Bioptic, Taiwan, China).

### Construction of the next generation sequencing library

Library construction and sequencing experiments were entrusted to Dian Diagnostics Group Co., Ltd. The initial amount needed to build a library was 200 ng DNA. We used Agencourt AMPure XP beads from Beckman Coulter in the United States to purify the DNA library. The Qubit 1×dsDNA Assay Kit (Thermo Fisher Scientific, Waltham, USA) was utilized to quantify the purified next-generation sequencing (NGS) library. Qsep100 (Bioptic, Taiwan, China) was used to analyze fragment size distribution. A panel of ten LUAD-related genes was used to determine the presence of single nucleotide variants, insertions, deletions, duplications, fusions, and delins mutations. LUAD-related genes include *EGFR, KRAS, ALK, ROS1, RET, MET, BRAF, HER2, PIK3CA* and *NRAS*. All experiments were conducted in a clinical laboratory improvement amendments-certified laboratory to ensure the genetic test’s quality.

### Sequencing and bioinformatics analysis

Illumina Nextseq 500 (Illumina, San Diego, USA) was used for library sequencing. The average sequencing depth was at least 1000X. The detection sensitivity of genetic variation was 1%. The FASTQ library’s paired-end sequencing data undergoes mapping to the human genome (hg19) through the Burrows-Wheeler Comparator (BWA-MEM) technology. The coverage depth of fusion breakpoints and adjacent sites were calculated by searching the possible fusion detection points. Somatic SNV was detected by muTect and somatic InDel by Strelka. Functional annotation of all the genetic variants was completed by ANNOVAR 21.

### Statistical analysis

For all analyses, we used R version 4.1.1 (2021–08–10). Continuous variables are usually reported as mean and standard deviations or median and interquartile ranges. The appropriate analytical tools for inter-group comparisons include the Student t-test or the Mann Whitney U-test. Subgroup analyses were evaluated using chi-square analysis, while Fisher’s exact test was used for small sample sizes. Kendall’s ratio was used for correlation analyses. The tests were two-sided, and significance was determined based on a criterion standard of P ≤ 0.05.

## Result

### Clinicopathological characteristics of patients

This study enrolled a cohort of 275 older LUAD patients, ranging in age from 61 to 88 years old, with a median age of 68 years old (**Supplementary Table S1**). There were 141 males and 134 females, with a male to female ratio of 1:1.05 and no sexual orientation. More than two-thirds of patients had no smoking history (199/275, 72.36%), and more than half of smokers had quit smoking (38/65, 58.46%). The incidence rate of the upper lobe was 1.89 times higher than that of the lower lobe (upper lobe 151 *vs*. lower lobe 80). Right lung incidence was 1.20 times higher than left lung incidence (right lung 148 *vs*. left lung 123). After imaging examination, multiple measurable lesions were found in 44 (16%) patients. Half of older LUAD patients were in Stage I. About one-fifth of patients experience lymph node metastasis. In addition, about one-fifth of patients experience involvement in pleural, mediastinal, bone, brain, and other metastases (**Table 1**).

**Table 1:** Demographic and clinical characteristics of 275 patients with LUAD.

Characteristics	No. of patients	%	Characteristics	No. of patients	%
**Age**	68 (61—88)		**No. of primary focus**		
**Gender**			Single lesion	231	84.00%
male	141	51.27%	Multiple lesions	44	16.00%
female	134	48.73%			
			**Tumor diameter (cm)**		
**Smoking history**			≦3	213	77.45%
no	199	72.36%	3> and≦5	47	17.09%
yes	27	9.82%	5> and≦7	15	5.45%
quit	38	13.82%			
unknown	11	4.00%	**Lymphatic metastasis**		
			**yes**	56	20.36%
**Location of lesions(pulmonary lobe)**			no	219	79.64%
upper lobe	151	54.91%			
lower lobe	80	29.09%	**Tumor metastasis**		
Upper&lower lobe	22	8.00%	yes	64	23.27%
unknown	22	8.00%	no	211	76.73%
**Location of lesions**			**Tumor stage**		
left	123	44.73%	Stage I	144	52.36%
right	148	53.82%	Stage II	10	3.64%
left&right	2	0.73%	Stage III	41	14.91%
unknown	2	0.73%	Stage IV	31	11.27%
			unknown	49	17.82%

### Gene mutation distributions and frequencies

**Figure 1:**
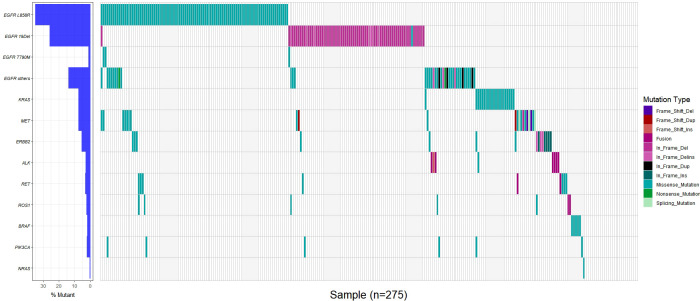
Genetic mutation in 275 geriatric LUAD patients.

In this study, the prevalence of genetic mutations in older LUAD patients was as high as 90.18% (248/275), whereas only a small minority of samples tested negative for mutations (27/275, 9.82%) (**Supplementary Table S2**). Among older LUAD patients with genetic mutations, 2 (2/248, 0.81%) patients had three driver gene mutations simultaneously, and 33 (33/248, 13.31%) had two co-gene mutations. Two hundred and thirteen (213/248, 85.98%) patients with only one driver gene mutation. It should be noted that a subset of patients (n = 22) exhibited only one driver gene mutation, but multiple mutation sites were present. For example, one patient displayed L858R, 19del, and S768I mutations in the EGFR gene. The results of the multi-gene analysis show that *EGFR* (192, 69.82%) had the highest mutation rate among ten genes, followed by *KRAS* (21, 7.64%), *MET* (21, 7.64%), *ERBB2* (15, 5.45%), *RET* (9, 3.27%), *ALK* (8, 2.91%), *ROS1* (8, 2.91%), *PIK3CA* (6, 2.18%), *BRAF* (5, 1.82%) and *NRAS* (1, 0.36%) (**Figure 1**).

**Figure 2:**
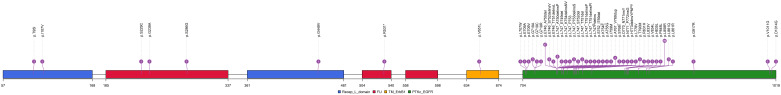
Distribution of mutations in EGFR functional domains.

Among 192 patients with *EGFR* mutations, 88.54% (170/192) patients had only single-site mutations. 10.94% (21/192) of patients had two mutations at the same time. Only one patient had three mutations in unison. The L858R mutation was identified in 50.00% of patients exhibiting *EGFR* mutations, while exon19 deletion was observed in 35.42% of patients with *EGFR* mutations. The two primary mutations found in *EGFR* were L858R and exon19 deletion, which account for 84.90% of patients with *EGFR* mutations. Rare *EGFR* mutations were found in 5 cases of *G719X*, 2 cases of *S768I*, and 3 cases of *L861R*. Three cases of classical drug resistance mutation *T790M* were detected. The average allele mutation frequency of *EGFR L858R* or *19del* carried by patients with *T709M* mutation (average allele frequency: 38.95%) was nearly twice that of patients without *T790M* mutation (average allele frequency: 19.56%). Fifty-one kinds of *EGFR* mutations were found, distributed in the protein tyrosine kinase catalytic domain (43, 84.31%), cysteine enriched domain (4, 7.84%), receptor binding domain (3, 5.88%), and EGFR transmembrane domain (1, 1.96%) (**Figure 2**). Thirty patients with *EGFR* mutations also experienced other gene mutations. One patient developed both *ROS1* and *RET* mutations, while the remaining patients had co-mutations with *MET, RET, ERBB2, ROS1, KRAS, ALK*, and *PIK3CA*, respectively (**Table 2**).

**Table 2:** EGFR genes incorporated mutations with other genes.

co-mutated gene	No. of patients	% EGFR mutation n=192	% LUAD n=275
EGFR+MET	10	5.21	3.64
EGFR+ERBB2	5	2.60	1.82
EGFR+PIK3CA	4	2.08	1.45
EGFR+RET	3	1.56	1.09
EGFR+ROS1	3	1.56	1.09
EGFR+ALK	3	1.56	1.09
EGFR+KRAS	1	0.52	0.36
EGFR+ROS1+RET	1	0.52	0.36

In addition, the fusion mutations were detected in 10 patients, including 5 cases of *ALK* fusion with two partner genes, *EML4* and *DCTN1* (**Supplementary Table S3**). In one case of *DCTN1/ALK*, the fusion type was *D24:A20*. In three cases of *ROS1* fusion, the fusion partner genes were *PKHD1 (P60:R34), EZR (E10:R34)* and *STK39 (R33:S11)*, respectively. Two fusion cases involving *RET* were identified: *KIF5B/RET (K15:R12)* and *RET/KCND2 (R11: K2)*.

### Associations between driver gene mutation and clinicopathological features in LUAD

**Figure 3:**
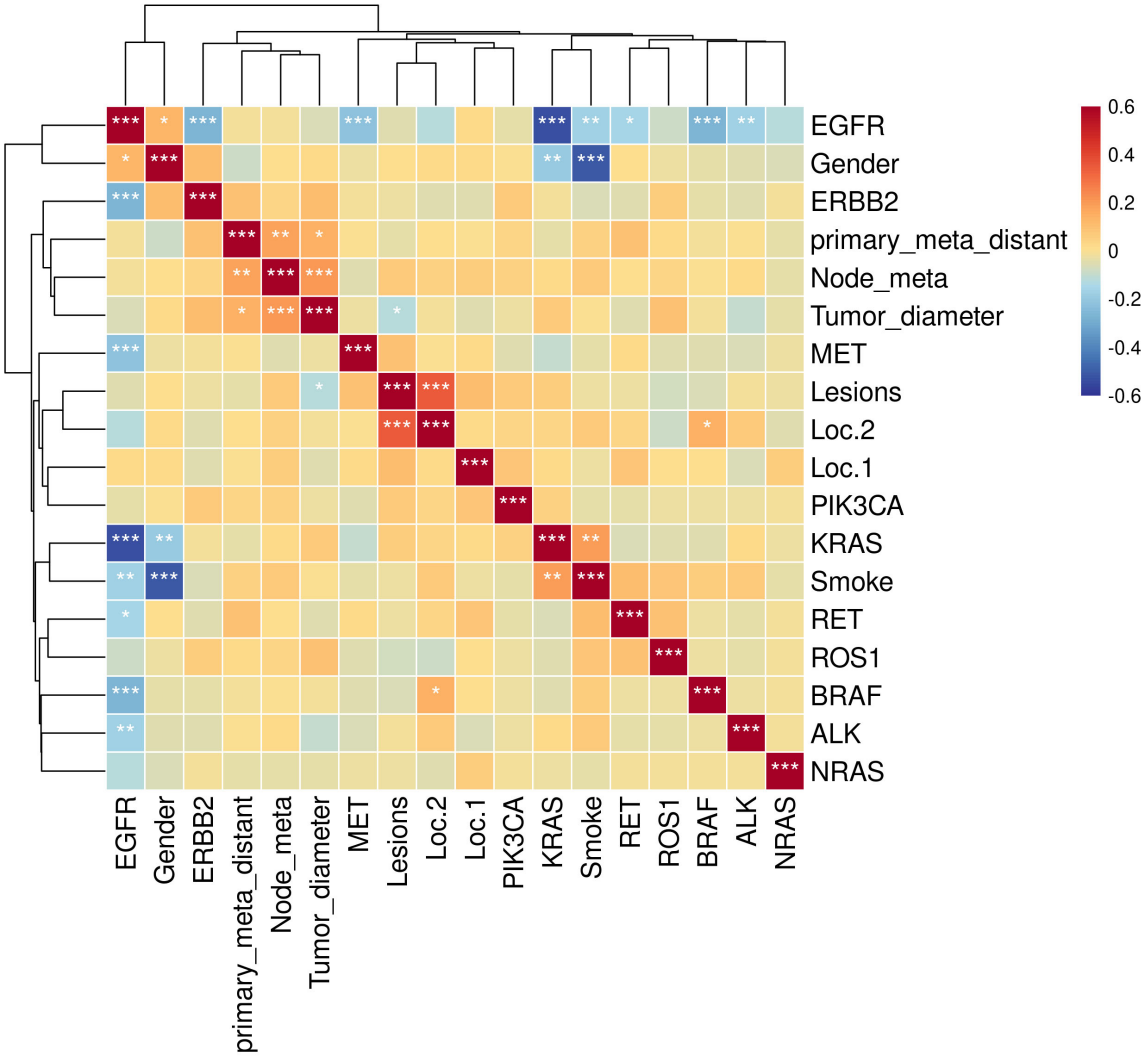
Kendall correlation analysis between gene mutations and clinical features. Test of significance of the Kendall correlation coefficient: “*”, “**”, and “***” represent p < 0.05, p < 0.01, and p < 0.001, respectively.

In older LUAD patients, female patients were more prone to genetic mutations than male patients (p = 0.0218). This feature was more pronounced in patients with *EGFR* mutations (p = 0.0040), though the potential significance were still unexplored (**Table 3**). No significant differences were observed among groups of older LUAD patients with different smoking histories regarding the occurrence of genetic mutations (p = 0.0965). However, the *EGFR* mutations were more likely to occur in patients without a history of smoking (p = 0.0024). No significant differences were observed in gene mutations or *EGFR* mutations based on lesion location, number of lesions, tumor size, lymph node metastasis, lesion metastasis and clinical stage. Kendall correlation significance test results showed that *EGFR* mutations were mutually exclusive with mutations in *MET, KRAS, ALK, ERBB2* and *BRAF* (**Figure 3**). In the correlation analysis, we discovered a noteworthy inverse correlation between *EGFR* mutation and smoking, as well as a significant positive correlation with gender (p < 0.01). It also mean that the *EGFR* mutations were more likely to occur in female and non-smoking patients. The *KRAS* mutation population distribution was opposite to *EGFR* mutation, which was significantly positively correlated with smoking (p < 0.01) and negatively associated with gender (p < 0.05). *BRAF* mutations were more likely to occur in the right lung (p < 0.05). In addition, the occurrence of metastasis in the primary lesion was positively correlated with tumor diameter (p < 0.05) and lymph node metastasis (p < 0.01).

**Table 3:** Comparison of gene mutations among different clinical indicator groups.

Characteristics	Negative	Gene mutation	P-value	Non-EGFR mutation	EGFR mutation	P-value
Gender						
male	20	121	0.0218	54	87	0.0040
female	7	127		29	105	
Smoking history						
no	15	184	0.0965	50	149	0.0024
yes	2	25		10	17	
quit	7	31		20	18	
Location of lesions(pulmonary lobe)						
upper lobe	19	132	0.1690	44	107	0.4317
lower lobe	4	76		30	50	
Upper&lower lobe	3	19		7	15	
Location of lesions						
left	12	111	0.8905	39	84	0.8489
right	15	133		43	105	
left&right	0	2		0	2	
No. of primary focus						
Single lesion	23	208	0.9207	68	163	0.6620
Multiple lesions	4	40		15	29	
Tumor diameter (cm)						
≦3	20	193	0.8654	61	152	0.5328
3> and≦5	5	42		16	31	
5> and≦7	2	13		6	9	
Lymphatic metastasis						
yes	5	51	0.9993	17	39	0.8957
no	22	197		66	153	
Tumor metastasis						
yes	5	59	0.7071	19	45	0.9545
no	22	189		64	147	
Tumor stage						
Stage I	14	130	0.931	45	99	0.8951
Stage II	0	10		2	8	
Stage III	4	37		12	29	
Stage IV	3	28		9	22	

## Discussion

The LUAD is the most common newly diagnosed malignant tumor in the older people (11, 12). As people age, older LUAD patients confront the dual challenge of declining organ function and heightened vulnerability to adverse treatment reactions (13). Previous clinical trials have found that the response rate to chemotherapy in older lung adenocarcinoma patients is only 9.9%-22.7% (14, 15). The response rate to gefitinib in LUAD patients with favorable *EGFR* mutations is approximately 70% (16, 17). Targeted therapies, which is guided by gene mutations, have significantly improved treatment choices and survival benefits in LUAD patients. In addition to common *EGFR* activation mutations, targets such as *ALK* rearrangement, *ROS1* rearrangement, *RET* rearrangement, *BRAF V600E*, *MET* exon 14 skip mutation, *KRAS G12C*, etc. have been gradually approved for targeted drug application in advanced non-small cell lung cancer (6, 18). However, Sabine Schmid, et al. (2) conducted a retrospective comparative analysis of the treatment benefits of older and young patients with advanced lung cancer over the past decade. The results showed that over the past ten years, compared to the significant improvement in 12-month cancer-specific survival for younger patients, there was only a slight improvement in older patients. While EGFR or ALK tyrosine kinase inhibitors (TKIs) therapy is typically more effective and well-tolerated than chemotherapy, the rate of genetic testing and subsequent targeted treatment among older patients remains relatively low. This may lead to a reduction in cancer-specific and overall survival rates in older patients. Our study retrospectively analyzed molecular detection results in 275 patients with lung adenocarcinoma over 60 years of age. The mutation frequency of *EGFR* gene in older LUAD patients was remarkably high at 69.82%. Key drug targets *EGFR L858R* and *19DEL* accounted for 84.90% of *EGFR* mutation patients. This indicates that more than four-fifths of patients may benefit from EGFR TKIs. Three patients carried *EGFR T790M* mutation and twenty-one patients developed *KRAS* mutation, providing reference information for drug selection. Five patients developed *ALK* fusion, including a rare fusion type *DCTN1/ALK (D24: A20)*. Gao Fangfang, et al. (19) have reported that one case of *DCTN1/ALK* achieved partial response after receiving cabozantinib therapy. *ERBB2, RET, ROS1, PIK3CA, BRAF*, and *NRAS* were detected in older LUAD patients. NGS-based multi-gene joint detection can provide more sensitive and comprehensive reference information for drug selection.

Molecular characteristics of LUAD are influenced by environmental factors, familial factor, and lifestyle factors (20, 21). Some studies emphasize that environmental factors such as age and smoking are closely related to the molecular characteristics of lung adenocarcinoma (22). Our analysis revealed a significant association between gene mutations and clinicopathological factors such as gender, smoking, and location of lesions. *EGFR* mutations were more likely to occur in female and non-smoking patients. Nevertheless, *KRAS* mutations were likely to occur in the male patient population (p < 0.01) and smokers (p < 0.01). *BRAF* mutations were more likely to occur in the right lung (p < 0.05).

There are several limitations to this study. This study was limited by the limited capacity of targeted sequencing panels, which may not reveal all mutations in older LUAD patients. Whole exome sequencing is a recommended methods for the comprehensive understanding of gene alterations. Furthermore, the study does not provide follow-up information on older LUAD patients. Therefore, we cannot analyze the correlation between molecular mutation characteristics and prognosis.

In conclusion, different molecular variations drive the occurrence and development of older LUAD patients. NGS can effectively expand our understanding about gene mutations and enable an integrated analysis of multiple gene mutations in older patients with LUAD, providing crucial evidence for targeted treatment.

## Availability of data and materials

The datasets generated and/or analyzed during the current study are available in the CNGB Nucleotide Sequence Archive (CNSA, https://db.cngb.org/cnsa/) repository with accession CNP0004388.

## Data availability statement

The datasets presented in this study can be found in the CNGB Nucleotide Sequence Archive (CNSA, https://db.cngb.org/cnsa/) repository with accession number CNP0004388 and can be found in the article/**Supplementary Material**.

## Ethics statement

The studies involving humans were approved by Medical Ethics Committee of the Affiliated Hospital of the Shandong University of Chinese Medicine (Ethical No. KY2023-081). The studies were conducted in accordance with the local legislation and institutional requirements. The ethics committee/institutional review board waived the requirement of written informed consent for participation from the participants or the participants’ legal guardians/next of kin because our institutional review board waived Informed consent because of the retrospective nature of our study.

## Author contributions

XL: Conceptualization, Project administration, Writing – original draft, Writing – review & editing. GJ: Data curation, Writing – review & editing. XS: Data curation, Writing – review & editing. GS: Data curation, Writing – review & editing. XZ: Formal Analysis, Methodology, Writing – review & editing. DS: Methodology, Writing – review & editing. NY: Conceptualization, Software, Writing – original draft, Writing – review & editing.
